# Improving Crop Yield through Increasing Carbon Gain and Reducing Carbon Loss

**DOI:** 10.3390/plants13101317

**Published:** 2024-05-10

**Authors:** Palanivelu Vikram Karthick, Alagarswamy Senthil, Maduraimuthu Djanaguiraman, Kuppusamy Anitha, Ramalingam Kuttimani, Parasuraman Boominathan, Ramasamy Karthikeyan, Muthurajan Raveendran

**Affiliations:** 1 Department of Crop Physiology, Tamil Nadu Agricultural University, Coimbatore 641003, India; karthickvikram555@gmail.com (P.V.K.); jani@tnau.ac.in (M.D.); niviani95@gmail.com (K.A.); kuttimanir@gmail.com (R.K.); boominathan.p@tnau.ac.in (P.B.); 2 Directorate of Crop Management, Tamil Nadu Agricultural University, Coimbatore 641003, India; karthikeyan.r@tnau.ac.in; 3 Directorate of Research, Tamil Nadu Agricultural University, Coimbatore 641003, India; raveendrantnau@gmail.com

**Keywords:** photosynthesis, Rubisco, photorespiration, CO_2_ concentrating mechanism

## Abstract

Photosynthesis is a process where solar energy is utilized to convert atmospheric CO_2_ into carbohydrates, which forms the basis for plant productivity. The increasing demand for food has created a global urge to enhance yield. Earlier, the plant breeding program was targeting the yield and yield-associated traits to enhance the crop yield. However, the yield cannot be further improved without improving the leaf photosynthetic rate. Hence, in this review, various strategies to enhance leaf photosynthesis were presented. The most promising strategies were the optimization of Rubisco carboxylation efficiency, the introduction of a CO_2_ concentrating mechanism in C_3_ plants, and the manipulation of photorespiratory bypasses in C_3_ plants, which are discussed in detail. Improving Rubisco’s carboxylation efficiency is possible by engineering targets such as Rubisco subunits, chaperones, and Rubisco activase enzyme activity. Carbon-concentrating mechanisms can be introduced in C_3_ plants by the adoption of pyrenoid and carboxysomes, which can increase the CO_2_ concentration around the Rubisco enzyme. Photorespiration is the process by which the fixed carbon is lost through an oxidative process. Different approaches to reduce carbon and nitrogen loss were discussed. Overall, the potential approaches to improve the photosynthetic process and the way forward were discussed in detail.

## 1. Introduction

The world’s population is expected to reach 10.4 billion by the end of this century [1]. The proportion of people suffering from hunger has slowly increased to half a billion globally, especially after the COVID-19 pandemic, which creates an urge to feed the world population. During the 1960s, the yield of major crops like rice and wheat increased due to the green revolution, which involved the use of high-yielding varieties, pesticides, fertilizers, and improved irrigation systems [2]. Over the last two decades, global stagnation in the annual increase in yield of major crops like rice, wheat, maize, and soybean has been observed despite agronomic and genetic advancements [3]. Major constraints of yield decline in major crops include deterioration of water and soil quality, increase in water consumption, and extinction of indigenous varieties of crops [4]. Apart from these, extreme and unpredictable weather conditions influence crop yield and the latest report of the Intergovernmental Panel on Climate Change (IPCC, 2023) stated that the current state of human-induced climate change is drastic, at least within the last 2000 years, and it is escalating in every region worldwide [5]. To address the rapid increase in demand for yield improvement, increasing photosynthesis could serve as a solution for improving plant productivity [6].

Photosynthesis includes a set of biochemical and biophysical mechanisms where the plants use sunlight and water to convert CO_2_ into sugars as well as to store carbohydrates, finally releasing O_2_ [7]. Over 90% of the CO_2_ transformed into biomass is utilized by the enzyme ribulose-1,5-bisphosphate carboxylase/oxygenase (Rubisco), constituting 30–50% of the soluble protein content in plant leaves. Even though Rubisco stands as one of the most ubiquitous proteins in nature, it is an inefficient enzyme with a low turnover rate, which makes it a constraint for photosynthetic carbon fixation under normal conditions [8]. Rubisco has the dual ability to react with Ribulose 1, 5-bisphosphate (RuBP) in the presence of both CO_2_ and O_2_. The carboxylation reaction forms two molecules of 3-phosphoglycerate (3-PG), which, under the Calvin cycle, is involved in the regeneration of RuBP. Similarly, the oxygenation reaction produces a single molecule of 3-phosphoglycerate (3-PG) and 2-phosphoglycolate (2-PG) [9]. The formed 2-PG is utilized in the process of photorespiration, which causes significant energy losses to the plants [10].

Generally, plants follow three different photosynthetic strategies to fix CO_2_ and they are classified as C_3_, C_4_, and CAM photosynthesis. In the metabolic pathway of C_3_ plants like rice, wheat, and soybean, Rubisco exerts a pivotal role in assimilating atmospheric CO_2_ [9]. However, during stressful conditions, this enzyme exhibits reduced affinity for distinguishing between O_2_ and CO_2_. Consequently, O_2_ competes with CO_2_ at the Rubisco active site, approximately resulting in a 50% loss of carbon from metabolic pathways. As a result, the photosynthetic efficiency of the C_3_ plants declines and other metabolic processes, such as photorespiration, intensify due to the oxygenase activity [11]. In plants like maize and sorghum with C_4_ photosynthetic pathway, CO_2_ is released close in vicinity to Rubisco, which favors more carboxylation than oxygenation. This assimilation mechanism serves to mitigate the potential loss of CO_2_ that typically occurs through photorespiration. Additionally, it also enhances water- and nitrogen-use efficiencies when compared to C_3_ plants [12]. Crassulacean acid metabolism (CAM) is found in plants that are grown under arid conditions. In this pathway, the stomata are found to be closed during the daytime to prevent evapotranspiration losses and opened at night time for the fixation of CO_2_ [13].

Most field crops like rice and wheat comprise the C_3_ metabolic pathway and are comparatively more inefficient than crops like maize and sorghum, consisting of the C_4_ metabolic pathway for CO_2_ uptake as well as assimilation [14]. The inefficiency of C_3_ plants is contributed by several factors like the slow turnover rate of Rubisco, less specificity of CO_2_ for Rubisco under stressful environments, increased photorespiration, and less CO_2_ availability at the site of fixation [9]. Therefore, to enhance the yield, the photosynthetic efficiency of these C_3_ plants primarily needs to be improved. In order to overcome the bottlenecks in C_3_ photosynthesis, various approaches like optimization of Rubisco and Calvin cycle enzymes, incorporation of CO_2_ concentrating mechanisms (CCM) into the plants, and alteration of photorespiration have been proposed [6]. This review briefly elaborates on the approaches to improve photosynthesis mainly focusing on the optimization of Rubisco, introduction of CO_2_ concentrating mechanisms (CCM), and manipulation of photorespiration.

## 2. Improving Photosynthesis by Optimizing Rubisco

Rubisco is an important regulating enzyme in the photosynthetic CO_2_ fixation pathway that functions for the fixation of 3–10 carbon atoms per second [15]. Due to this less carboxylation efficiency, enhancing the catalytic properties of Rubisco becomes an essential target for increasing photosynthesis [16].

Rubisco, a protein complex, is made of 16 subunits that comprise 8 large (RbcL) and 8 small (RbcS) subunits [17]. The formation of Rubisco in eukaryotic organisms is a complex process that entails contributions from both genomes of the nucleus and chloroplast. Specifically, the smaller RbcS subunits are encoded by genes of the nucleus, while genes of chloroplasts encode the larger RbcL subunits [18]. The primary sequence of these subunits controls Rubisco’s functional characteristics. Various chaperones encoded by the nucleus assist in the folding and assembly of Rubisco and Rubisco activase (RCA), which aids in activating the inactive Rubisco enzymes [19]. These chaperones are found to be the key regulators of controlling the formation of Rubisco. The manipulation of these factors serves as a possible mechanism for enhancing Rubisco’s activity.

### 2.1. Modification of Rubisco Chaperones

Rubisco folding and assembling is an important process that is regulated by a group of proteins called Rubisco chaperones. Hsp70 is the primary chaperone that is found to be responsible for the interaction with the newly synthesized protein peptide of the larger subunit RbcL, facilitating its initial folding within the chloroplast [19]. Subsequently, the folded protein chain is transferred to the Cpn60 (Chloroplast Chaperonin 60) chaperone units, comprising Cpn60α and Cpn60β subunits [20]. Additionally, Cpn10 and Cpn20 are identified as facilitators of Cpn60s, aiding in the folding of RbcL in plants [21]. Conversely, the peptide of the smaller subunit RbcS, formed in the cytoplasm, undergoes folding and translocation into the chloroplast for assembly [22]. Following the folding of the subunits, the assembly of the Rubisco holoenzyme commences, facilitated by assembly chaperones such as the Rubisco accumulation factor (Raf1 and Raf2) and bundle sheath defective 2 (BSD2) [23]. These chaperones aid in stabilizing the RbcL dimer initially, leading to the synthesis of an RbcL_8_ core through the binding of four chaperone-associated RbcL dimers. Subsequently, these chaperones are eventually replaced by RbcS to form a Rubisco L_8_S_8_ complex [19].

Considering the importance of these chaperones in the formation of Rubisco, the effectiveness of Rubisco’s carboxylation activity is dependent upon its proper assembly and stability. Increased levels of Raf1 caused by the increased expression of Raf1 can lead to a significant enhancement in the content of Rubisco. In maize bundle sheath cells, overexpression of Raf1, along with RbcL and/or RbcS, resulted in a notable increase of over 30% in Rubisco content [24]. This enhancement may be attributed to the excessive accumulation of Raf1, which serves to stabilize the complex consisting of Rubisco assembly proteins and shield the subunits of Rubisco from degradation [25]. Salesse-Smith et al. [24] suggested that the inability of Rubisco assembly to result in the aggregation of Rubisco catalytic units within mesophyll cells may be attributed to the absence of the chaperones that assist in the assembly of Rubisco and the instability of RbcL and RbcS transcripts in these cells. However, a recent investigation demonstrated the successful assembly of Rubisco within maize chloroplasts by co-expressing RbcL, RbcS, Raf1, Raf2, and BSD2 proteins [26]. Similarly, a research study aimed at the primary construction of Rubisco from *Arabidopsis* in *Escherichia coli* indicated that the production of a fully functional Rubisco necessitated the co-expression of all factors (Raf1, Raf2, BSD2, and RbcX) alongside the chloroplast chaperonin system [27].

Biogenesis of the photosynthetic enzyme Rubisco is a complex enzyme having eight large and eight small subunits that require an assembly chaperone for Rubisco catalysis. Rubisco biogenesis in plastids can be modified by transplastomic approaches by expressing the Arabidopsis Rubisco large subunits and tobacco Rubisco accumulation factor 1 (Raf1) in tobacco [28]. The presence of the AtRaf1 chaperone led to a two- to three-fold increase in the amount and biogenesis rate of hybrid Rubisco in tobacco leaves compared to the control, resulting in a two-fold increase in the photosynthetic CO_2_ assimilation rate and plant growth (Table 1). Therefore, the modification of these chaperones can be considered an approach to improve Rubisco synthesis and enhance the Rubisco content, ultimately increasing the photosynthetic rate of the crops.

### 2.2. Alteration in Rubisco Subunits

Even though the fundamental roles of Rubisco remain consistent within the photosynthetic pathway, the catalytic efficiency shows variability across species as variations influence it in the Rubisco subunits. Harnessing different types of Rubisco sources, each with unique abilities to catalyze and bind CO_2_, could enhance plant photosynthesis and improve the efficiency of C_3_ photosynthesis [39]. An approach involves the development of a hybrid Rubisco through the replacement of either larger or smaller units of native Rubisco with more effective subunits sourced from different species. This aims to enhance the carboxylation capacity of Rubisco in C_3_ plants [40].

A study in rice utilizing a hybrid Rubisco composed of RbcS subunits from C_4_ sorghum combined with RbcL subunits from rice demonstrated increased catalytic activity and enhanced CO_2_ assimilation rates, particularly in high irradiation environments [41]. This is possibly due to the alteration in Rubisco kinetics that played an important role in enhancing the CO_2_ carboxylation rate. Conversely, in another study, an L_8_S_8_ hybrid Rubisco, created by introducing RbcL from sunflower Rubisco into tobacco chloroplasts, exhibited similar catalytic characteristics and stability [42]. Nevertheless, transgenic plants experienced a reduction in CO_2_ assimilation rates due to insufficient levels of foreign mRNA of larger subunit RbcL and the instability of sunflower larger subunit assembly with smaller tobacco subunits [42]. A research investigation found that incorporating C_4_ *Flaveria bidentis* RbcL into tobacco led to a notable enhancement in the catalytic rate of the hybrid Rubisco. However, this increase did not correspond to a rise in carboxylation efficiency and a decrease in CO_2_ affinity was observed [16].

Rubisco’s eight small subunits are coded by the nuclear multigene family (RBCS). Genetic modification of Rubisco content has been proved by the transformation of antisense RBCS construct in tobacco. Transgenic tobacco expressing antisense RBCS had lower levels of RBCS mRNA and normal levels of Rubisco large subunit coding gene (RbcL). However, the amount of RbcL protein was adjusted according to the RBCS protein level at the RbcL protein translation initiation process. Suzuki et al. [29] overexpressed the RBCS gene, which did not improve photosynthesis because the RBCS protein level maintains the Rubisco level. In another study, overexpression of RBCS and RbcL did not increase the Rubisco content [24]. However, overexpression of RBCS, RbcL, and Rubisco assembly chaperone 1 increased the Rubisco protein content, resulting in an increased photosynthetic rate. The results indicate that Rubisco biogenesis requires molecular chaperones like cylindrical chloroplast chaperonin, Cpn60, and its cofactor Cpn20 for the folding of RbcL. Also, the folded RbcL requires Rubisco-specific assembly chaperones, namely RbcX, Raf1 and 2, and the Bundle sheath defective-2 (BSD2), to mediate the assembly of the RbcL_8_ intermediate complex. Transgenic maize plants overexpressing RbcL, RBCS, and Raf1 showed a 15% increase in CO_2_ assimilation, which increased the fresh weight. Also, the rate of photosynthesis is dependent on Rubisco activase (Table 1).

An alternative approach to increase the rate of carbon assimilation is overexpressing RbcL from lower organisms because it can reduce the complexity of Rubisco biogenesis. Hence, RbcL from *Rhodospirillum rubrum* was expressed in tobacco through a transplastomic approach. The result indicated that the transgene did not increase the photosynthetic rate as evidenced by a slow growth rate [43]. Similarly, Form 1A Rubisco derived from the proteobacterium *Halothiobacillus neapolitanus* can successfully assemble functional L_8_S_8_ hexadecamers in tobacco chloroplasts [44]. However, the effects were not observed under ambient CO_2_ concentrations. However, other forms of Rubisco from lower organisms can improve the photosynthetic process [45]. This approach opens up promising avenues for integrating Rubisco activity from diverse species into chloroplast photosynthetic metabolism. However, Rubisco engineering encounters challenges associated with simultaneously modifying genes encoding Rubisco subunits across different plant genomes.

Although the relatively enhanced catalytic efficiency was observed in certain studies through the integration of exogenous Rubisco or its subunits, notable limitations have become evident. These drawbacks have resulted in either stagnant or reduced biomass in transgenic plants, thereby constraining the practical application of such techniques for enhancing photosynthesis. Challenges arise from the low affinity between these subunits, possibly arising from difficulties in assembling these hybrid Rubisco subunits RbcL and RbcS. Therefore, ensuring high compatibility for the successful assembly of Rubisco subunits from diverse species becomes imperative. Moreover, future efforts should prioritize the development of Rubisco enzymes with heightened CO_2_ affinity alongside maintaining high catalytic rates.

### 2.3. Manipulation of Rubisco through Rubisco Activase

The activation of Rubisco relies on the involvement of Rubisco activase (Rca), a regulatory protein essential for the process. Rubisco activase facilitates the removal of inhibitors from Rubisco’s active sites, thereby enhancing carbamylation [46]. Rubisco activase functions as an ATPase, employing the energy derived through ATP hydrolysis to displace inhibitors from Rubisco’s active sites, facilitating its activation [47]. Under normal conditions and high light intensity, Rubisco is typically activated to levels ranging from 80% to 90%, with the exception being at high temperatures. Evidence has substantiated the conclusion that the decline in Rubisco activity due to heat primarily starts from the temperature-dependent inactivation of Rubisco activase [48]. Rubisco activase displays remarkable sensitivity to heat-induced inactivation, with its optimal temperature for the activity being below 40 °C [49]. Enhancing the thermal stability of Rubisco activase could yield advantages for Rubisco functionality, particularly demonstrating more potential under elevated temperatures [50]. In a research study conducted by Yamori et al. [51], it was demonstrated that the thermal stability of photosynthesis experienced a slight improvement through the overexpression of maize Rubisco activase along with the native rice Rubisco activase. Considering the optimal temperature for photosynthesis in these plant varieties, the introduced maize Rubisco activase is expected to share a similar temperature preference with the native Rubisco activase in rice. Scafaro et al. [52] reported that the overexpression of a Rubisco activase with enhanced thermal stability sourced from wild rice into cultivated rice was found to enhance grain yield. Hence, to improve the Rubisco carbamylation process, it is mandatory to enhance the Rca. The genetic diversity of Rca [53] and its response to elevated temperature has to be explored to improve the crop yield. In this context, desert plants will be the potential sources of Rca because these plants are exposed to a temperature higher than 50 °C [54,55]. Inconsistent with the above hypothesis, the β-isoform of agave Rca showed activity up to 52 °C, whereas rice Rca lost its activity at 45 °C itself. Analysis of rice-agave Rca chimeras indicated that the trait of thermostability was associated with the N-terminal 244 residues of the agave protein.

Fukayama et al. [30] validated the above hypothesis by developing transgenic rice by incorporating the Rubisco activase from barley or maize, a crop that has higher temperature tolerance than rice. The transgenic rice expressing barley or maize Rca showed high levels of expression and enhanced Rubisco’s activation state. However, the CO_2_ assimilation rate was substantially decreased in transgenic lines, possibly due to post-transcriptional mechanisms. However, there was no evidence for reduced transcript levels of RBCS or RbcL in transgenic lines (Table 1). However, through gene shuffling technology, several thermostable RCA1 variants were generated and then introgressed into an *Arabidopsis* RCA deletion line [31] and the thermostable RCA1 variants showed higher photosynthetic rates. The same concept was proved in rice by overexpressing Rca. At high temperatures, the transgene overexpressing Rca had a higher photosynthetic rate than the control, which could be due to decreased Rubisco deactivation at high temperatures [32]. In maize, two forms of Rca were observed (α-form and β-form) [34]. In a comprehensive analysis of 123 maize inbred lines, both RCA forms exhibited a positive correlation with grain yield, showing the association observed with three C_4_ photosynthesis genes (Table 1).

The impact of overexpressing rice Rca in maize on photosynthesis was studied by Feng et al. [37]. The transgenic lines exhibited an elevated Rubisco activity and activation state, leading to higher net photosynthetic rates and enhanced PSII photochemical quantum yield compared to wild-type plants (Table 1). In conclusion, the introduction of Rubisco activase, which is stable under higher temperatures, is essential to mitigate the temperature-dependent deactivation of Rubisco carboxylation and photosynthesis. Further progress in the development of thermostable Rubisco activase paves the way for increasing photosynthesis through modification of Rubisco activity.

In summary, optimizing the Rubisco through the manipulation of chaperones and subunits serves as a possible strategy to improve the Rubisco’s activity. The chaperone proteins play a crucial role in the folding and assembly of Rubisco subunits. The overexpression of these chaperones could enhance the Rubisco content, thereby increasing the CO_2_ assimilation rate. The Rubisco subunits could be manipulated to create a hybrid Rubisco by combining the native Rubisco with another exogenous Rubisco. Even though the hybrid Rubisco had higher catalytic efficiency, the biomass of the transgenic plants remained either constant or reduced and this needs to be focused on in the future. Apart from these, modifying the thermal stability of Rubisco activase serves as a key opportunity to increase photosynthesis, especially in a changing climate scenarios.

## 3. Introduction of a CO_2_ Concentrating Mechanism (CCM) for Enhancing Photosynthesis

The integration of CO_2_ concentrating mechanisms (CCMs) into C_3_ plant species has been found to be a pivotal force of numerous synthetic biology approaches that are focused on enhancing photosynthesis efficiency [56]. Increasing the concentration of CO_2_ around the active Rubisco of C_3_ plants can be achieved through CO_2_ concentrating mechanisms (CCMs). These mechanisms are found in C_4_ and CAM plants as well as in cyanobacteria and algae [8]. The C_4_ plants are characterized by the presence of Kranz anatomy, where the vascular bundle sheath cells are surrounded by mesophyll cells, which serve as a CO_2_-concentrating mechanism that facilitates more carboxylation. In C_4_ plants, during the process of photosynthesis, the conversion of phosphoenolpyruvate (PEP) occurs in both mesophyll and bundle sheath cells. This transformation, along with continuous regeneration facilitated by transport mechanisms, results in the production of inorganic carbon at a concentration approximately 10 times higher than that of Rubisco [57]. Hence, the integration of the C_4_ cycle into C_3_ plants serves to be an appropriate approach to improve the CO_2_ concentration around Rubisco. Upregulating the genes related to maize into rice has been shown to increase the accumulation of photosynthetic enzymes, potentially enabling the transformation of C_3_ plants into C_4_ plants [58]. Additionally, upregulating the expression of maize transcription factor GLK in rice has been demonstrated to improve photosynthetic efficiency [59]. Increasing the expression of enzymes involved in the Calvin cycle significantly contributes to enhancing photosynthesis. The effects of overexpressing maize phosphoenolpyruvate carboxylase (PEPC) cDNA in *Arabidopsis thaliana* were studied by Kandoi et al. [33]. The developed transgenic lines exhibited significantly higher PEPC protein content and activity, resulting in increased levels of four-carbon carboxylic acids like malic acid and oxaloacetate. The increased levels of these carbon skeletons facilitated enhanced amino acid and protein synthesis, which ultimately contributed to the metabolic processes such as chlorophyll biosynthesis and photosynthesis (Table 1). Similarly, Driever et al. [35] focused on augmenting the levels of sedoheptulose-1,7-biphosphatase (SBPase) enzyme in wheat. Transgenic wheat lines were developed through genetic transformation and expression of SBPase gene constructs from *Brachypodium distachyon*. These lines exhibited increased photosynthesis through higher SBPase protein levels and activity, which finally resulted in enhanced yield (Table 1). Even though numerous studies were carried out conceptually to explain the engineering of C_4_ photosynthesis into C_3_ plants, improvement is still needed for field-level testing of such transgenic plants.

The CAM plants temporally separate the functions of phosphoenolpyruvate carboxylase (PEPC) and ribulose-1,5-bisphosphate carboxylase/oxygenase (Rubisco), rather than spatially as seen in C_4_ plants [60]. In CAM species, CO_2_ fixation occurs primarily during the night when stomata are closed, which reduces evaporation and enhances water use efficiency (WUE), thereby making it an advantageous trait for arid conditions [61]. Hence, these transgenic C_3_ plants that have been engineered with the CAM pathway offer a promising target for synthetic biology approaches aimed at enhancing WUE in C_3_ crops, particularly under warmer and drier conditions [62]. These mechanisms constitute the biochemical type of CO_2_ concentrating mechanisms.

Another category of biophysical CO_2_ concentrating mechanisms includes those found in cyanobacteria and algae. In algae such as *Chlamydomonas*, CO_2_ is uptaken as bicarbonate (HCO_3_^−^), which is converted to CO_2_ in the vicinity of Rubisco. Concentrating CO_2_ directly within cells of algae imposes challenges as it is a small uncharged molecule that facilitates rapid diffusion across membranes. To overcome this issue, CO_2_ is taken as bicarbonate (HCO_3_^−^). In order to optimize the efficiency of CO_2_ fixation, Rubisco is enclosed within a specialized microcompartment known as the pyrenoid, located within the chloroplast [63]. The *Chlamydomonas* model of CCM proposed by Fei et al. [64] reveals two types of modes of operation differing only in the way of HCO_3_^−^ accumulation. The first one is the passive type, where CO_2_ passively diffuses from the periplasm through the plasma membrane via the low CO_2_ inducible one channel (LCI1) [65]. Subsequently, it traverses the chloroplast envelope to enter the chloroplast stroma through low CO_2_ inducible B (LCIB). In the active type, CO_2_ undergoes conversion to HCO_3_^−^ facilitated by the carbonic anhydrases CAH1 and CAH2 [66]. HCO_3_^−^ then traverses the plasma membrane through the transporter high light activated 3 (HLA3) and subsequently accumulates across the chloroplast envelope mediated by low CO_2_ inducible A (LCIA). Within distinct pyrenoid-traversing domains of the thylakoid membranes, carbonic anhydrase 3 (CAH3) facilitates the conversion of HCO_3_^−^ to CO_2_, a process stimulated by the acidic pH of the thylakoid lumen [67]. CO_2_ molecules diffuse outward from the thylakoid membranes and enter the pyrenoid matrix, where Rubisco sequesters them. It is hypothesized that a barrier preventing CO_2_ leakage slows down the escape of CO_2_ from the pyrenoid, resulting in heightened CO_2_ concentration and reduced energy expenditure [64]. Transferring the carbon concentrating mechanism (CCM) from *Chlamydomonas* into higher plant species entails the introduction of various components into multiple cellular compartments and membranes. Additionally, there may be a need to eliminate native carbonic anhydrase and aquaporins to prevent interference with the CCM process. Initial investigations have shown promising outcomes, with key *Chlamydomonas* CCM proteins—such as HLA3, LCIA, and LCIB—successfully localizing to their intended locations in higher plants [68]. Furthermore, there have been successful instances of stable functional expression of *Chlamydomonas* Rubisco in *Arabidopsis*, indicating progress in implementing CCM components across plant species [68]. However, achieving a fully operational carbon concentrating mechanism (CCM) may necessitate the aggregation of Rubisco molecules into a pyrenoid-like structure. Currently, the exact number of components required for pyrenoid assembly remains uncertain.

The introduction of a reaction-diffusion model focused specifically on the *Chlamydomonas* chloroplast and provided insights into the components and the energetic expenses that are associated with developing a functional pyrenoid-based carbon concentrating mechanism (CCM) [64]. This model identified three crucial parts that are essential for enhancing CCM efficacy. The first part included the development of a carbon dioxide (CO_2_) uptake mechanism in chloroplasts either by a stromal carbonic anhydrase to facilitate the diffusion of CO_2_ into bicarbonate ions (known as the LCIB-dependent pathway) or by an active pump, which could import the bicarbonate ions (HLA3/LCIA-mediated HCO_3_^−^ uptake). The second part focused on the transportation of bicarbonate ions into the thylakoid lumen. This could be made possible through the channels on the thylakoid membrane (BSTs). Lastly, the presence of a condensed Rubisco within the pyrenoid matrix, enveloped by diffusion barriers that restrict the leakage of CO_2_, was made essential for enhanced carboxylation [69]. Configurations of the CCM that lacked or disrupted any of these three modules were found to incur an increased ATP requirement per assimilated CO_2_ as well as diminished capacity to concentrate inorganic carbon.

Similarly, in cyanobacteria, Rubisco is enclosed within specialized protein-based microcompartments called carboxysomes, which serve as localized sites for increased CO_2_ concentrations [70]. These carboxysomes typically range in size from 90 to 400 nanometers in diameter with icosahedral structures and are composed of 20 equilateral triangular facets. They hold either a significant portion or the entire part of the active Rubisco enzyme within the cell [71]. Two distinct lineages of carboxysomes are present, which are denoted as α and β. These lineages emerged independently in freshwater cyanobacteria (β lineage) as well as in the marine cyanobacteria (α lineage) [72]. Despite the variations in gene organization and protein sequences, both lineages have a similar carboxysome structure and functionality. Under typical conditions, individual unicellular cyanobacterial cells generally contain 5–15 carboxysomes, with the exact number varying based on the species and growth environment. The essential process of CO_2_ supply within these microcompartments relies heavily on the catalytic activity of a carboxysome-localized carbonic anhydrase (CA) [73]. This enzyme, referred to as CcaA or CcmM, depending on the specific type of carboxysome, facilitates the conversion of accumulated cytosolic HCO_3_^–^ into CO_2_. Here, the CO_2_ is taken up with the help of various bicarbonate transporters and converted to bicarbonate by carbonic anhydrase found near the Rubisco in carboxysomes. The proteinaceous shell of the carboxysome is found to possess a unique ability to inhibit the leakage of CO_2_ in comparison to the movement of ionic forms during the process of entry and exit [74]. Additionally, CO_2_ pumps situated within the thylakoid membrane are instrumental in the process of reutilizing CO_2_ that escapes from the carboxysome, effectively returning it to the HCO_3_^−^ pool.

Considerable advancements have been made in an attempt to synthesize carboxysomes within the chloroplasts of C_3_ plants, with tobacco serving as a primary model system [75]. Nevertheless, a significant challenge lies in the initial step necessary for integrating a functional biophysical CCM into C_3_ plants, which necessitates ensuring the expression and accurate localization of CO_2_ transporters from cyanobacteria or green algae into C_3_ plants. The development of functional carboxysomes, which are capable of concentrating CO_2_, particularly in alternative host organisms, has been a significant objective in bioengineering [76]. However, the fact that the structure alone cannot fully replicate the CCM has made this goal challenging [77]. Initially, recombinant carboxysomes were produced in *Escherichia coli* by expressing the α-carboxysome related gene from the model proteobacterium *Halothiobacillus neapolitanus.* The expression of the native 10-gene operon was found to be adequate for generating carboxysomes resembling the wild-type [78]. Similarly, a synthetic operon of 12 genes from cyanobacterium *Synechococcus elongatus* PCC7942 produced β-carboxysomes similar to wild type in *E. coli* [79]. The engineered carboxysomes in both studies possessed active Rubisco but it remained unclear whether the recombinant carboxysomes could concentrate carbon, which is the essential feature of carboxysome. A mutagenesis screening of *Halothiobacillus neapolitanus* under high CO_2_ and low CO_2_ conditions revealed dozens of new genes responsible for the functioning of α-carboxysome CCM, including several proteins that are uncharacterized [80]. Recent work by Long et al. [81] demonstrated the successful production of simplified carboxysomes within tobacco chloroplasts, wherein the endogenous Rubisco large subunit genes were replaced with cyanobacterial form-1A Rubisco large and small subunit genes. This innovation resulted in the formation of carboxysomes that could concentrate Rubisco, thereby facilitating enhanced growth at elevated CO_2_ levels. These findings present the alternative pathways for refining the microcompartment design based on simplified genes.

In conclusion, the introduction of biophysical and biochemical CCM into C_3_ plants serves as one of the approaches to improve photosynthesis. Further research is needed to focus on understanding the structural and organelle differentiation in C_4_ photosynthesis to engineer it in C_3_ crops. Detailed elucidation of the core key components of microcompartments of the biophysical CCM in cyanobacteria and algae is needed for engineering the C_3_ plants with these structures to increase CO_2_ concentration around Rubisco for improved carboxylation efficiency.

## 4. Utilization of Photorespiratory Bypasses for Improving Photosynthetic Efficiency

Photorespiration is the biochemical process wherein RuBP interacts with O_2_ in the presence of Rubisco, resulting in the production of 2-phosphoglycerate (2-PG) and 3-phosphoglycerate (3-PG). Due to the substantial carbon loss caused by the photorespiratory pathway, redesigning the pathway emerges as a significant strategy for enhancing photosynthetic efficiency [82]. Various engineered photorespiratory bypass pathways have been developed in C_3_ plant species, including *Arabidopsis*, tobacco, and rice. These pathways primarily target the metabolism and recycling of 2-phospho- glycolate (2-PG) produced through RuBP oxygenation, with the aim of reducing glycine decarboxylation and limiting ammonia release in mitochondria. This approach seeks to minimize the loss of metabolic compounds and the energy spending related to ammonia re-fixation [82].

Under the first bypass, the five protein-encoding genes of three glycolate dehydrogenase (GDH) subunits (D, E, and F), glyoxylate carboligase (GCL), and tartronic semialdehyde reductase (TSR) were isolated from *Escherichia coli* and introduced into *Arabidopsis* [83]. These constituted the bacterial glycolate pathway in *Arabidopsis*, which facilitated the direct conversion of glycolate into glycerate without the formation of ammonia. This reduction in the emission of ammonia decreases the consumption of ATP and reduces equivalents. The plants transformed with this pathway showed greater photosynthetic rates and lower photorespiration [83]. A novel strategy was introduced by Nölke et al. [38] to enhance photosynthetic carbon fixation in potatoes (*Solanum tuberosum*). In this strategy, a polyprotein (DEFp), which contains three subunits (D, E, and F) of *Escherichia coli* glycolate dehydrogenase (GlcDH) was expressed in potatoes. The engineered polyprotein functioned similar to the native GlcDH complex, which is present in *E. coli*. Transgenic potato plants started to accumulate DEFp in plastids, where the recombinant protein exhibited increased activity, reducing photorespiration and enhancing CO_2_ uptake, thereby significantly impacting carbon metabolism. In the second bypass mechanism reported by Carvalho et al. [84], the GCL and hydroxy pyruvate isomerase (HYI) genes were introduced into the peroxisome of the tobacco plant for the direct conversion of glyoxylate to hydroxy pyruvate without the emission of toxic ammonia. As the expression of hydroxy pyruvate isomerase (HYI) in tobacco failed, the conversion of glyoxylate to hydroxy pyruvate was not possible. Maier et al. [85] proposed the third bypass mechanism where the external malate synthase (MS) from *Cucurbita maxima* and catalase (CAT) from *E. coli* introduced into *Arabidopsis* chloroplast along with its innate glycolate oxidase converted the glycolate into CO_2_ completely. This study found higher photosynthesis and dry matter production only under low light conditions, which makes it unsuitable for crops like rice that require high light intensity.

The fourth bypass was found in tobacco chloroplasts, incorporating malate synthase (MS) from *Cucurbita maxima* and glycolate dehydrogenase (GDH) from *C. reinhardtii* to mitigate H_2_O_2_ production. By confining glycolate conversion to CO_2_ and its release solely inside the chloroplast, the exit of glycolate generated through photorespiration was curtailed through the silencing of the glycerate transporter of plastids PLGG1 [86] and this can increase the CO_2_ concentration around the Rubisco enzyme. In another approach, Shen BoRan et al. [36] and Shen BoRan et al. [36] proposed a GOC bypass in rice, in which three native rice genes encoding glycolate oxidase (OsGLO3), oxalate oxidase (OsOXO3), and catalase (OsCAT) were expressed into the chloroplasts. This relocation aimed to fully oxidize the glycolate generated under photorespiration, leading to the release of CO_2_ within the chloroplasts of rice [36] and increased biomass and yield were recorded under field and greenhouse conditions.

In the GCGT bypass (sixth bypass) that was built in rice, the genes encoding proteins of *E. coli* catalase (EcCAT), glyoxylate carboligase (EcGCL), and tartronic semialdehyde reductase (EcTSR) were introduced into the rice [87]. These enzymes, along with rice glycolate oxidase (OsGLO1), produced the metabolites oxalate and glyoxylate, which initiated distinct reactions, resulting in the complete oxidation of all glycolate into CO_2_ with its subsequent return to the Calvin cycle, similar to native photorespiration in the GOC bypass. However, in the GCGT bypass, only 75% of glycolate was oxidized into CO_2_. Transgenic rice lines utilizing the GOC and GCGT bypasses exhibited notable improvement in photosynthesis as well as yield. Moreover, certain plants demonstrated characteristics similar to those grown in environments abundant in CO_2_, indicating elevated photosynthetic rates, increased sugar and chlorophyll content, excess chloroplast size in leaves, and greater accumulation of starch grains [88].

**Figure 1:**
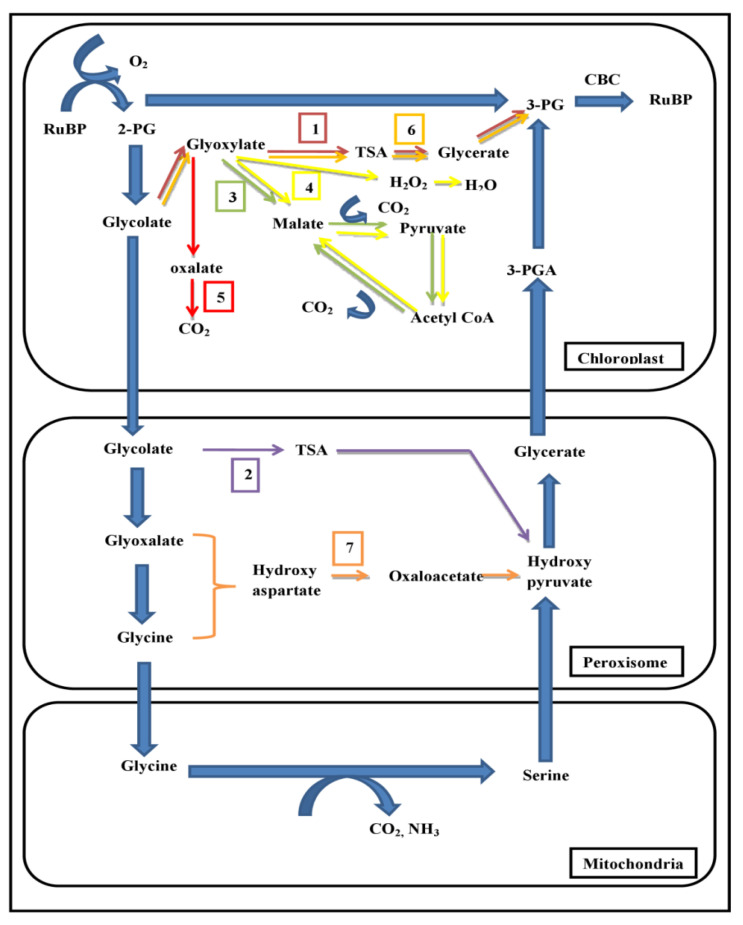
Various photorespiratory bypasses are designed in plants. Different colors indicate the photorespiratory bypass pathways (1–7). CBC—Calvin Benson Cycle; TSA—Tartronic Semialdehyde; 3-PG—Phosphoglycerate; RuBP—Ribulose-1,5- Bis Phosphate.

While existing photorespiratory bypasses have shown improvements in photosynthesis and biomass accumulation, they release CO_2_ within organelles, potentially leading to carbon leakage and reduced efficiency compared to natural photorespiration [89]. The β-hydroxy aspartate cycle (BHAC) in proteobacteria of marine ecosystems offers benefits due to its carbon-conserving nature [90]. In this cycle, glyoxylate is directly converted into oxaloacetate (OAA) with no loss of carbon through a four-step enzymatic process. An attempt to engineer a photorespiratory bypass based on the BHAC was made in *Arabidopsis*, involving the introduction of genes encoding these enzymes [91]. This bypass functioned in plant peroxisomes under photorespiratory conditions and showed nitrogen efficiency. Integration of the BHAC into synthetic C_4_ photosynthesis pathways in C_3_ plants, starting with OAA without PEPC-catalyzed CO_2_ fixation, holds promise for achieving higher yields despite challenges related to 3-phosphoglycerate regeneration and metabolic conversion of OAA. Furthermore, the introduction of bypasses into C_3_ plants presents unresolved challenges, leading to limitations in the application of these approaches. Figure 1 depicts the various photorespiratory bypass pathways (1–7) that are discussed above.

In summary, the photorespiratory bypasses discussed above can be utilized to reduce the carbon losses caused by photorespiration. Even though they are successfully integrated with C_3_ plants, limitations exist that prevent yield performance under field conditions. In another way, the integration could also limit the function of existing carbon metabolism, thus affecting its practical application. Hence, the unanticipated impacts of introduced bypasses denote the need for adaptive regulation of additional metabolic pathways when implementing novel bypass strategies.

Various approaches have been utilized to increase photosynthetic efficiency. Table 1 summarizes various approaches followed in crops for enhancing photosynthesis.

## 5. Conclusions and Future Perspectives

Enhancing crop yields sustainably is imperative to fulfill the increasing needs of a growing global population for food, feed, and various plant-based goods. Therefore, optimizing photosynthetic efficiency emerges as a critical element for ensuring food security amidst anthropogenic climate changes and constraints on resources. In the pursuit of enhancing crop productivity by enhancing photosynthetic efficiency in C_3_ plants, strategies such as optimizing the Rubisco enzyme, introducing CO_2_ concentrating mechanisms (CCM), and manipulation using photorespiratory bypasses were developed and also tested in model plants as well as crops like rice. Despite notable advancements, unresolved queries like the relationship between abiotic stresses and photosynthesis remain, which must be elucidated, which is crucial for fully harnessing the potential enhancements attainable through these methodologies. Głowacka et al. [92] studied the influence of chloroplast size on photosynthesis and it was observed that the manipulation of chloroplasts did not improve photosynthetic rate.

In the optimization of Rubisco, apart from improving its catalytic efficiency, many factors such as optimizing its assembly and folding need to be considered in Rubisco engineering. The Rubisco subunits of the C_3_ plants can be enhanced by replacing them with subunits of more efficient C_4_ crops to improve the Rubisco, with due importance given to its assembly and folding for its proper activity. Enhancing Rubisco carboxylation activity in C_3_ crops also demands the upregulation of the Rubisco activation state, which requires the expression of more active Rubisco activases (RCAs) rather than just overexpressing the exogenous Rubisco holoenzyme.

Conversely, under the photorespiration pathway, numerous questions persist regarding the enhancement of photosynthetic efficiency through modification of the photorespiration process. A significant portion of the transport proteins involved in this process remains uncharacterized. Hence, there is a need to identify novel components of the photorespiratory pathway in order to elucidate the complete photorespiratory cycle. While numerous synthetic photorespiratory bypasses are being developed in C_3_ plants, the efficacy of these created pathways remains undetermined. Key aspects such as carbon and nitrogen flow traversing along the bypasses and the impact of metabolic reorganization on other pathways of various metabolisms remain obscure and need to be elucidated. Apart from these, various studies conceptually explain the engineering of C_4_ photosynthesis into C_3_ plants but a focus is still needed for the field-level development of such transgenic plants.

On the other hand, for the successful engineering of CCMs into these C_3_ plants, detailed knowledge of the core components of these biophysical and biochemical CCMs is required to be elucidated. Synthetic biology can be exploited for the introduction of the C_4_ pathway into C_3_ plants to improve photosynthesis. The introduction of artificial intelligence, synthetic biology, gene editing, and other novel strategies holds immense scope for uncovering novel pathways to optimize photosynthesis. By synergistically integrating rapid RuBP carboxylation, increased CO_2_ concentration around Rubisco enzymes, and lowered energy usage for photorespiration, researchers can attain increased photosynthesis and crop productivity.

## Figures and Tables

**Table 1:** Approaches to improve photosynthesis in plants.

Crop	Approach	Impact	Reference
Tobacco	Overexpression of *Arabidopsis* Rubisco chaperone RAF1 and subunit in Tobacco	Improvement in Rubisco content and activity was found along with more photosynthetic rate	Whitney et al. [28]
Rice	Increasing the expression of Rubisco subunit	Enhances the Rubisco content as well as higher photosynthetic rate	Suzuki et al. [29]
Maize	Overexpression of Rubisco subunit	23% Decrease in Rubisco activation state due to Rubisco activase limitation	Salesse-Smith et al. [24]
Rice	Overexpression of Rubisco activase	Decrease in Rubisco content as well as photosynthetic rate	Fukayama et al. [30]
*Arabidopsis*	Increasing the thermal stability of Rubisco activase	Increases the photosynthetic rate	Kurek et al. [31]
Rice	Overexpression of both Rubisco and Rubisco activase under heat stress	Increases photosynthetic rate and yield	Qu et al. [32]
*Arabidopsis*	Overexpressing C_4_ specific Phosphoenolpyruvate carboxylase	Reduced Photorespiration	Kandoi et al. [33]
Maize	Characterization of Rubisco activase isoforms (α-form and β-form)	Enhances the photosynthetic rate	Yin et al. [34]
Wheat	Increasing Seduheptulose—1,7—Bis Phosphatase (SBPase) activity by overexpression	Enhanced photosynthetic rate, biomass and yield	Driever et al. [35]
Rice	Introduction of GOC photorespiratory bypass	Increase in photosynthetic rate and nitrogen content with reduction in photorespiration	BoRan et al. [36]
Maize	Rubisco Activase Overexpression	Improved Rubisco content, activity and photosynthetic rate.	Feng et al. [37]
Potato	Genetic modification to express Glycolate Dehydrogenase (GDH)	Increased CO_2_ assimilation rate and biomass accumulation	Nölke et al. [38]
